# High Prevalence of Vitamin D Deficiency in Native *versus* Migrant Mothers and Newborns in the North of Italy: A Call to Act with a Stronger Prevention Program

**DOI:** 10.1371/journal.pone.0129586

**Published:** 2015-06-11

**Authors:** Francesco Cadario, Silvia Savastio, Corrado Magnani, Tiziana Cena, Veronica Pagliardini, Giorgio Bellomo, Marco Bagnati, Matteo Vidali, Erica Pozzi, Stella Pamparana, Mauro Zaffaroni, Giulia Genoni, Gianni Bona

**Affiliations:** 1 Division of Pediatrics, Department of Health Sciences, University of “Piemonte Orientale Amedeo Avogadro”, Novara, Italy; 2 IRCAD (Interdisciplinary Research Center of Autoimmune Diseases), Novara, Italy; 3 Unit of Medical Statistics and Cancer Epidemiology, Department of Translational Medicine, University of “Piemonte Orientale Amedeo Avogadro” and CPO-Piemonte, Novara, Italy; 4 University of Turin, Turin, Italy; 5 Central Laboratory of Maggiore della Carità Hospital, University of “Piemonte Orientale Amedeo Avogadro”, Novara, Italy; University of São Paulo, BRAZIL

## Abstract

**Background:**

Vitamin D status during pregnancy is related to neonatal vitamin D status. Vitamin D deficiency has been associated with an increased risk of rickets in children and osteomalacia in adults. Aim of this study was to investigate 25OHD levels in maternal serum and in neonatal blood spots in native and migrant populations living in Novara (North Italy, 45°N latitude).

**Methods and Findings:**

We carried out a cross sectional study from April 1st 2012 to March 30th 2013, in a tertiary Care Center. Maternal blood samples after delivery and newborns' blood spots were analyzed for 25OHD levels in 533 pairs. Maternal country of origin, skin phototype, vitamin D dietary intake and supplementation during pregnancy were recorded. Multivariate regression analysis, showed a link between neonatal and maternal 25OHD levels (R-square:0.664). Severely deficient 25OHD values (<25 nmol/L) were found in 38% of Italian and in 76.2% of migrant’s newborns (p <0.0001), and in 18% of Italian and 48,4% of migrant mothers (p <0.0001) while 25OHD deficiency (≥25 and <50 nmol/L) was shown in 40.1% of Italian and 21.7% of migrant’s newborns (p <0.0001), and in 43.6% of Italian and 41.3% of migrant mothers (p <0.0001). Italian newborns and mothers had higher 25OHD levels (34.4±19.2 and 44.9±21.2nmol/L) than migrants (17.7±13.7 and 29.7±16.5nmol/L; p<0.0001). A linear decrease of 25OHD levels was found with increasing skin pigmentation (phototype I 42.1 ±18.2 vs phototype VI 17.9±10.1 nmol/l; p<0.0001). Vitamin D supplementation resulted in higher 25OHD values both in mothers and in their newborns (p<0.0001).

**Conclusions:**

Vitamin D insufficiency in pregnancy and in newborns is frequent especially among migrants. A prevention program in Piedmont should urgently be considered and people identified as being at risk should be closely monitored. Vitamin D supplementation should be taken into account when considering a preventative health care policy.

## Introduction

Vitamin D is an anti-rachitic agent and its deficit is the leading cause of rickets in children and osteomalacia in adults. Moreover, in recent years, vitamin D deficiency has been considered as a possible cause of several diseases, including microbial infections, cardiovascular and allergic diseases, cancers, type 1 diabetes, multiple sclerosis and other autoimmune diseases [1–10].

Vitamin D levels during fetal life and the neonatal period may play a role in the development of these conditions through epigenetic changes [11], therefore, vitamin D status during pregnancy and in the newborn could provide important information.

The aim of this study was to assess the vitamin D status in a population of mothers, living in Novara (Piedmont, North Italy, 45° parallel of latitude), at the time of delivery and in their newborns two days after birth. Italians and migrants from emerging countries were studied in order to investigate possible critical deficiencies and risk factors in the two groups.

## Subjects and Methods

### Study design and population

The investigation was performed in Novara, Piedmont (North Italy, 45° latitude), in a tertiary Center of Obstetrics and Neonatology. We enrolled Italian and migrant mothers after delivery, with their newborns. We included mothers and newborns in pairs after uncomplicated pregnancies who underwent vaginal or surgical delivery.

All healthy newborns at term (37–41 weeks) appropriate for gestational age (AGA) according to Italian charts [12] were eligible for the study.

Neonatal exclusion criteria were: Apgar score <7 at 1’ and < 8 at 5’ minutes, gestational age < 37 weeks, newborn small or large for gestational age (SGA-LGA), signs of distress at birth, intra partum complications, any congenital disease, and neonatal jaundice. Maternal exclusion criteria were: gestational diabetes, presence of any psychiatric or organic disease (renal failure, liver failure, other endocrine diseases) and any particular pharmacological treatment (corticosteroids, antiepileptic drugs, immunosuppressants) (S1 Strobe checklist).

Birth weight and length were recorded at birth by the attending nurse. Mothers’ weight at delivery and before pregnancy were also recorded.

The Ethical Committee of the “AOU Maggiore della Carità”, Novara, approved the study protocol and all mothers signed an informed consent. A translator was consulted in case of language difficulties.

The goal was to consecutively enrol 40 newborns with their mothers every month from the corresponding set of eligible subjects: 20 from Italian mothers and 20 from migrant mothers. However, it was not possible to reach the quota every month for migrants due to different responses to informed consent and to the lower percentage of migrants.

Data collection lasted one year, starting from April 1st 2012 until March 30th 2013, to avoid seasonality bias.

Interview-administered questionnaires were employed post-partum, assessing data on maternal ethnicity, education, clinical history and health behaviour. Vitamin D intake through diet during pregnancy was investigated with EPIC Italy semiquantitative questionnaires and analyzed with an electronic system [13].

Data on maternal vitamin D supplementation during the whole pregnancy and during the last month of pregnancy, including doses, duration and type of integration were collected.

We considered, and analyzed as adequately supplemented all mothers with an intake of 400 IU vitamin D per day as recommended by The Institute of Medicine [14].

Mothers were evaluated for countries of origin (Italy, North Africa, Central-Southern Africa, East Europe, Asia, South America), and skin phototypes according to Fitzpatrick Skin type chart [15].

### Vitamin D measurements

We rated vitamin D levels (nmol/L) in neonatal blood spots (NBS) collected in the first three days of life for neonatal metabolic screening. Vitamin D concentrations were measured by a tandem mass spectroscopy (TripleQuad6410 and LC/MS/MSsu Agilent: HPLC Infinity1290) in a sample of 3 mm punches from dried blood spots; to minimize the impact on newborns a drop was texted contextual with screening for metabolic diseases. The variability coefficient (CV) for inter-assay analyses was 13.4%. Data were reconverted to serum concentration in nmol/L with an algorithm that took into consideration the neonatal hematocrit [16–17].

We analyzed 25OHD serum levels (nmol/L) of mothers within three days after delivery with a direct competitive chemiluminescent immunoassay (Liaison Test 25OHD total, DiaSorin Inc, Stillwater MN-USA). Blood samples were collected post-partum during routine analysis. CV for inter-assay analyses was 10%.

NBS values measured by LC-MS and maternal values measured by immunoassay methods were comparable after reconverted neonatal vitamin D levels to serum concentration (nmol/L) using the same algorithm [16, 18–21].

Vitamin D values were graded as insufficient <75 nmol/L, deficient <50 nmol/L and severely deficient < 25 nmol/L as per the Endocrine Society [22]. The analysis was also performed considering as sufficient vitamin D levels ≥50 nmol/l, as insufficient levels between 25 nmol/L and 50 nmol/L, and as deficient levels <25 nmol/L, as recommended by The Institute of Medicine [14].

### Statistical analysis

Data were expressed as mean ± standard deviation (SD) or percentages as appropriate. For continuous variables, differences among Italians and migrants were compared using parametric T Student Test (T test) and χ2 test for categorical variables. Each variable predictive or associated with 25OHD levels was analyzed with univariate regression. Multiple regression analysis was used to estimate the strength of association among variables which were statistically significant in univariate analysis. The multivariate analysis model for neonatal and maternal 25OHD levels was developed adding one-by-one the potential factors implicated, starting from the variables most strongly associated in univariate analyses, discarding at each step the non-significant factors or the newly inserted variable if not significant to alter the estimates of previously included factors (neonatal/maternal 25OHD levels, ethnicity, seasonality). Phototype and ethnicity were almost co-linear variables and for this reason they could not be analyzed together in the models.

The “ethnicity” variable (in mothers and newborns) was univocal and easier to identify than phototype which was assigned according to Fitzpatrick Skin type chart. Vitamin D supplementation and dietary intake were analyzed only in maternal models because they closely correlated. Statistical significance was defined by p values <0.05. All statistical analyses were performed using SAS for Windows version 9.2.

## Results

**Fig 1:**
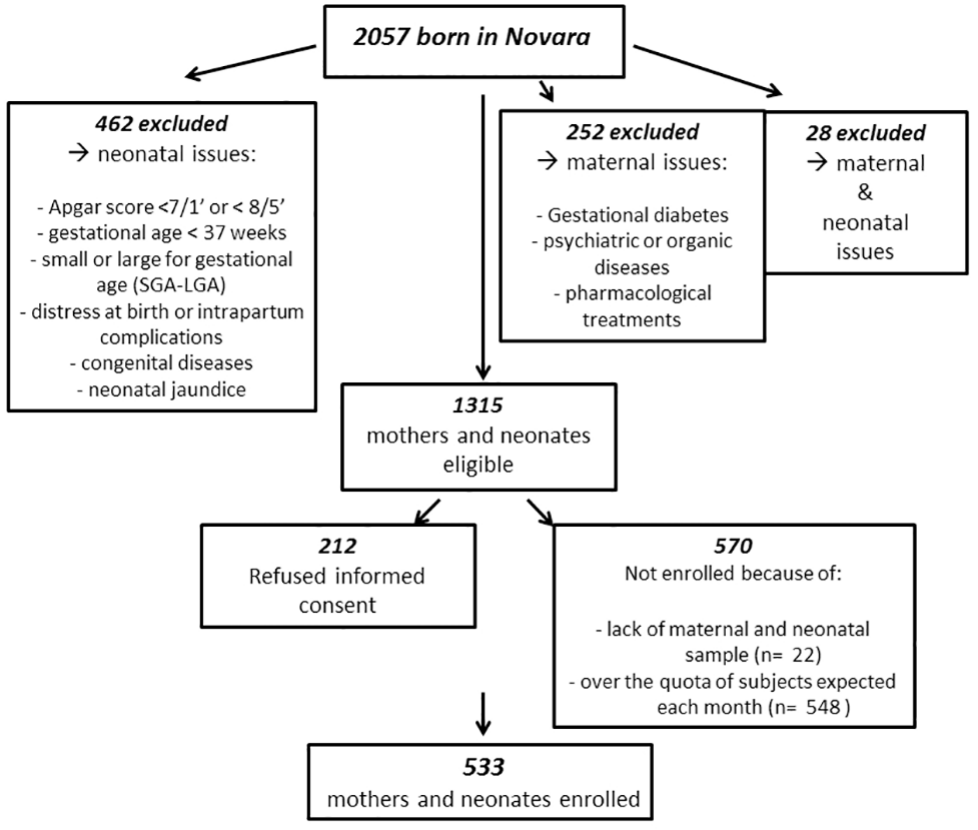
Flowchart of the enrolled subjects.

From April 1st 2012 to March 30th 2013, 533 mothers and newborns were included in our analysis from a total of 2057 deliveries at our Hospital (Fig 1).

Among enrolled participants, 342 (64.2%) were Italians and 191 (35.8%) were migrants: 52 (27.2%) from North Africa (Algeria, Egypt, Morocco, Tunisia), 48 (25.1%) from East Europe (Albania, Bosnia, Moldova, Poland, Romania, Ukraine), 39 (20.4%) from Asia (Bangladesh, Syria, Turkey, Pakistan, Philippines, China, Sri Lanka), 23 (12%) from Central-South America (Brazil, Bolivia, Venezuela, Caribbean), 29 (15.2%) from Central-South Africa (Ghana, Ivory Coast, Nigeria, Senegal, Togo).

Italian newborns had a lower birth weight than migrants (p<0.02). Birth length and mode of delivery were not different between Italian and migrant newborns. Migrant mothers were younger than Italian (p<0.0001). Education levels and weight gain during pregnancy were higher in Italian mothers (p<0.0001 and p<0.02, respectively) (Table 1).

**Table 1:** Characteristics of study population.

	**Newborns**			
	**All (533)**	**Italian (342)**	**Migrant (191)**	**p value**
**Birth weight (g)**	3330.4±365.1	3273.6±359	3348.5±372	0.022
**Birth length (cm)**	50±1.8	50±1.8	50.2±1.8	0.141
**Natural delivery (%)**	341 (63.9%)	214 (62.57%)	127 (66.49%)	0.366
**Surgical delivery (%)**	192 (36.02%)	128 (37.43%)	64 (33.51%)	0.366
	**Mothers**			
	**All (533)**	**Italian (342)**	**Migrant (191)**	**p value**
**Age (years)**	32.3±5.6	33.6±5	29.8±5.8	<0.0001
**Weight before (Kg)**	61.4±13	60.8±13.5	62.5±12	0.211
**Weight delivery (Kg)**	73.6±12.5	73.2±12.8	74.5±11.8	0.324
**Weight gain (Kg)**	11.8±5.1	12.2±4.9	11.2±5.4	0.021
**PT 1–2 (%)**	106 (20.8%)	92 (28.7%)	14 (7.5%)	<0.0001
**PT 3–4 (%)**	284 (55.9%)	214 (66.9%)	70 (37.2%)	<0.0001
**PT 5–6 (%)**	118 (23.2%)	14 (4.4%)	104 (55.3%)	<0.0001
**Primary school licence**	18 (3.4%)	0 (0.0%)	18 (9.7%)	<0.0001
**Junior high school certificate**	145 (27.7%)	71 (21.0%)	74 (39.8%)	<0.0001
**High school diploma**	208 (39.7%)	144 (42.6%)	64 (34.4%)	<0.0001
**Graduation**	153 (29.2%)	123 (36.4%)	30 (16.1%)	<0.0001

Data are expressed as mean±SD and number (%).

PT: maternal phototype

According to the Endocrine Society criteria, we found severely deficient 25OHD values (<25 nmol/L) in 38% of Italian and 76% of migrant newborns (p <0.0001), and in 18% of Italian and 48,8% of migrant mothers (p <0.0001). Analyzing only migrant subjects, both newborns and mothers had vitamin D deficiency (<50 nmol/L) respectively in 97.9% and 89.7%. Considering values ≥50 nmol/l as vitamin D sufficiency (IOM criteria), among newborns, 33.3% showed insufficiency and 52.1% a deficiency. Mothers had vitamin D insufficiency in 42.9% and deficiency in 27.7%. Migrant’s newborns and mothers had a vitamin D deficiency in 76.2% and 48.4%, respectively.

The predominant percent concentration of 25OHD values for both Italian babies and mothers was between 25 and 50 nmol /L, while for migrant babies and their mothers it was below 25 nmol /L (Table 2).

**Table 2:** Vitamin D levels (25OHD; nmol/l) in newborns and mothers overall and according to seasonality (Endocrine criteria).

	**Newborns**			
**25OHD levels (nmol/L)**	**All (533)**	**Italian (342)**	**Migrant (191)**	**p value**
**x≥75 n (%)**	11 (2.1%)	10 (3.1%)	1 (0.5%)	<0.0001
**50≤x<75 n (%)**	64 (12.5%)	61 (18.8%)	3 (1.6%)	<0.0001
**25≤x<50 n (%)**	171 (33.3%)	130 (40.1%)	41 (21.7%)	<0.0001
**x<25 n (%)**	267 (52.1%)	123 (38%)	144 (76.2%)	<0.0001
**25OHD levels (nmol/L)**	28.2±19.2			
**Winter/spring**	20.7±14.7	27.2±14.9	17.4±11.9	<0.0001
**Summer/autumn**	34.1±20.2	43.9±18.9	23.2±14.9	<0.0001
	**Mothers**			
**25OHD levels (nmol/L)**	**All (533)**	**Italian (342)**	**Migrant (191)**	**p value**
**x≥75 n (%)**	36 (7.5%)	33 (10.1%)	3 (1.9%)	<0.0001
**50≤x<75 n (%)**	106 (21.9%)	93 (28.3%)	13 (8.4%)	<0.0001
**25≤x<50 n (%)**	207 (42.9%)	143 (43.6%)	64 (41.3%)	<0.0001
**x<25 n (%)**	134 (27.7%)	59 (18%)	75 (48.4%)	<0.0001
**25OHD levels (nmol/L)**	39.9±21			
**Winter/spring**	32.4±16.4	37.6±16.4	31.2±15.4	0.0019
**Summer/autumn**	45.4 ±22.2	54.9±21.4	34.9±17.2	<0.0001

Data are expressed as frequencies (number %) or as mean±SD.

**Fig 2:**
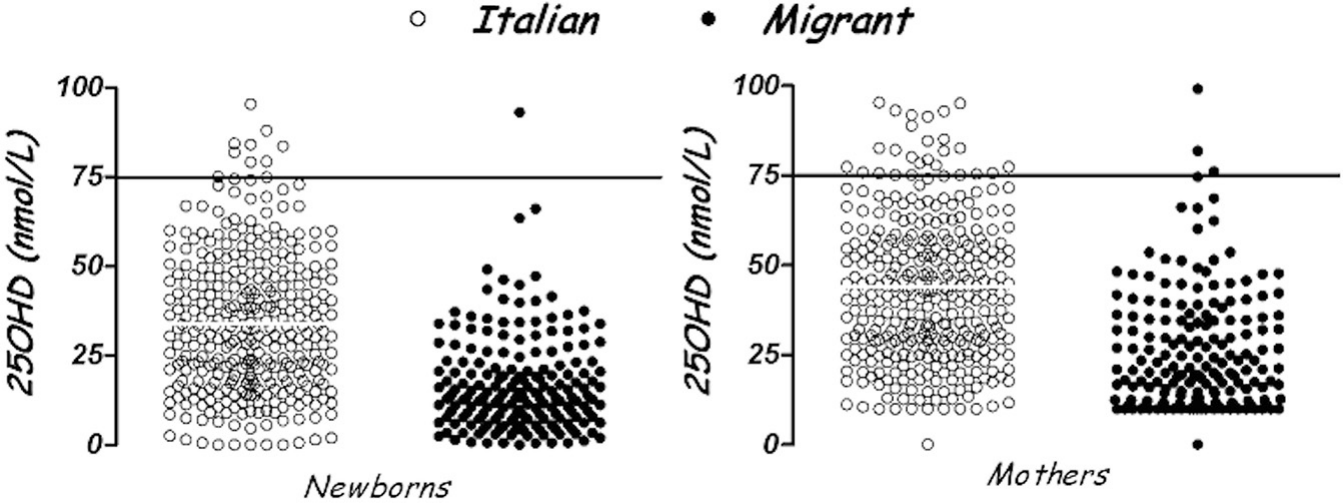
Vitamin D (25OHD) levels (nmol/L) in Italian and migrant mothers and newborns. * = p<0.0001

25OHD levels were (mean±SD) 28.2±19.2 nmol/L in neonatal blood spots and 39.9±21 nmol/L in maternal serum. Levels were higher in Italian mothers than migrant (p<0.0001) and in Italian newborns (p<0.0001) (Table 2) (Fig 2).

**Fig 3:**
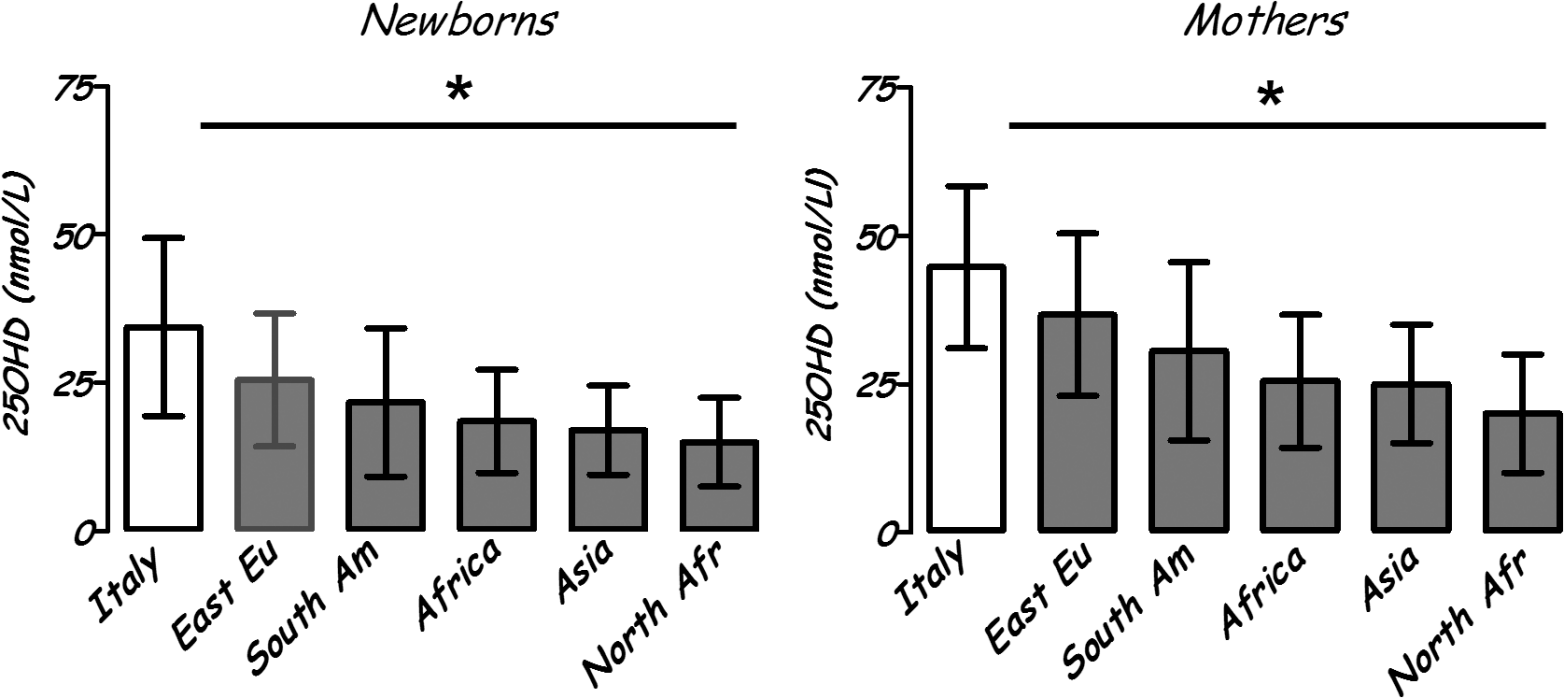
Vitamin D (25OHD) levels (nmol/L) in mothers and newborns by country of origin (mean ± SD). * = p<0.0001

Analyzing data by country of origin, Italian newborns had 25OHD levels higher than all ethnic groups (p<0.0001) (North African, African, Asian, Central-South American and East European). The same results were found in mothers. North African mothers and newborns had the lowest 25OHD levels (respectively, 21.7±10.5 and 12.7±10 nmol/L) (Fig 3).

Mothers and newborns with phototype V or VI had 25OHD values (respectively, 30.2±19.2 and 17.9±10.1 nmol/l) lower than subjects with phototype I or II (42.1±18.2 and 32.6±19.7 nmol/l; p<0.0001) and phototype III or IV (41.4±21.2 and 30.2±18.7 nmol/l; p<0.0001).

Analyzing data by seasonality, 237 (44.5%) mothers and newborns were enrolled in winter-spring and 296 (55,5%) in summer-autumn without significant differences. 25OHD levels were lower in winter-spring than in summer-autumn in mothers and newborns both Italians and migrants (p<0.0001) (Table 2).

Data on vitamin D supplementation and dietary intake in pregnancy are reported in Table 3.

**Table 3:** Maternal vitamin D supplementation and dietary intake during pregnancy in Italian and migrants.

Supplementation	All (533)	Italian (342)	Migrant (191)	p value
**n (%)**	283/486 (58.2%)	213 (68.9%)	70 (39.5%)	<0.0001
**Last month n (%)**	255/483 (52.8%)	193 (62.9%)	62 (35.2%)	<0.0001
**Duration of supplementation (days)**	185.7±75.14	191.8±74.6	167.6±74.4	0.01
**Vitamin D intake from supplementation (IU/die)**	398±52	400.47 ±61.19	394.37±44.36	0.01
**Diet questionnaires analyzed n (%)**	450/533 (84.4%)	298 (66.2%)	152 (33.8%)	-
**Vitamin D intake from diet (IU/die)**	136±68	128±64	152±68	0.0003

Data are expressed as frequencies or as mean±SD and number (%).

All mothers ‘supplemented’ had used a daily prenatal multivitamin complex containing 10 mcg (400 IU) of vitamin D3 in 88% of cases (250/285), 12,5 mcg (500 IU) in 7% (21/285) or 5 mcg (200 IU) in 5% (14/285). The average daily vitamin D intake was 398±52 IU/d. Not all mothers regularly took the supplements and the percentage of women ‘supplemented’ was further reduced in the last month of pregnancy both in Italian and migrants.

All mothers ‘supplemented’ with vitamin D had 25OHD values higher than ‘not supplemented’ (p<0.0001) and the same was recorded in their newborns (p<0.0001) (Table 4).

**Table 4:** Comparison between vitamin D levels (25OHD) in newborns and mothers supplemented or not supplemented with vitamin D.

**25OHD levels (nmol/l)**	**S**	**Not S**	**p value**
**Newborns**	33.9±19.7	19.4±14.9	<0.0001
**Mothers**	44.6±20.7	29.7±16.7	<0.0001
**25OHD levels (nmol/l) in the last month**	**S**	**Not S**	**p value**
**Newborns**	34.6±19.7	19.4±14.9	<0.0001
**Mothers**	45.4±20.4	29.7±16.7	<0.0001

Data are expressed as mean±SD.

S: subjects supplemented with vitamin D; Not S: subjects not supplemented with vitamin D

Analyzing EPIC Italy semiquantitative questionnaires we found an average daily vitamin D intake of 136±68 UI (3.4 ± 1.7 μg) with a higher intake in migrant mothers than Italian (p = 0.0003) (Table 3).

A daily supplementation of 400 IU of vitamin D led to 105 (37%) mothers (93 Italian and 12 migrants) and 56 (20%) newborns (53 Italian and 3 migrants) to have blood 25OHD values above the cut-off of sufficiency (fixed at 50 nmol/L) (Table 5).

**Table 5:** Number and percentage of subjects under vitamin D supplementation (400 IU per day) showing sufficient levels of vitamin D (25OHD≥50 nmol/L).

Subjects	All (283)	Italian (213)	Migrant (70)	p value
**Newborns**	56 (20%)	53 (24.9%)	3 (4.3%)	<0.001
**Mothers**	105 (37%)	93 (43.7%)	12 (17.1%)	<0.001

No significant associations between maternal and neonatal 25OHD levels and maternal weight gain in pregnancy or birth weight were found. Multivariate adjusted regression model analysis (R-square: 0.302), adjusted for maternal age and education showed a link between maternal 25OHD level and season (p<0.0001), country of origin (North Africa, p<0.0001; Africa, p = 0.0052; Asia, p = 0.0003; South America, p = 0.0002) and vitamin D supplementation in pregnancy (p<0.0001), while vitamin D intake from diet was non-significant (p = 0.944).

A multivariable regression model analysis (R-square: 0.664) showed a link between the dependent variable neonatal 25OHD level and the independent variable maternal 25OHD level (p<0.0001) adjusted for country of origin, season, maternal age and birth weight.

## Discussion

A high prevalence of vitamin D deficiency has been reported among newborns and their mothers worldwide [23–27]. Our study showed a high prevalence of 25OHD deficiency in a population of mothers-newborns living in Novara (Piedmont, North Italy, 45° latitude), especially among migrants, according to IOM and Endocrine criteria.

These two classifications of vitamin D status are significantly divergent and the issue of desirable vitamin D levels for a given age is a matter of great debate, especially in the light of the skeletal and extraskeletal effects of vitamin D (these were not been investigated in this study).

In our study, North African mothers and newborns had the lowest 25OHD values; this finding is in line with previous data suggesting that ethnic origin is a risk factor for vitamin D deficiency [23–24]. Vitamin D is synthesized in the skin through exposure to 290–315 nm ultraviolet light or by dietary intake. Sunlight exposure, latitude, seasons and skin colour are the main determinants of 25OHD status. Individual variables such as indoor lifestyle, head covering, poor dietary intake and lack of supplementation can led to deficiency; furthermore previous studies have reported that genetic factors contribute to variability in 25OHD levels, with estimates of heritability ranging between 28.8% and 43% in different populations [28–30]. These studies even if limited to specific populations (Caucasians [28], Arab an Asian [29], rural populations of South China [30]) show a complex picture of genetic factors determining the level of 25OHD and consequent risks of deficiency of vitamin D. Despite this, the environmental component is still preponderant although limited to 57–71% in determining the level of 25OHD.

In our study the defect 25OHD appears to be related to immigration per se rather than to a single ethnicity and this could be primarily linked to environmental factors such as sunlight exposure, latitude, lifestyle and diet.

As already known, placental transfer provides vitamin D from mother to fetus, especially in the last trimester of gestation [31] thus neonatal and maternal 25OHD levels are closely linked.

However, we found lower 25OHD levels in infants compared to their mothers. Moreover, in a previous study, we found lower vitamin D levels on cord blood than in maternal serum [26]. The mechanisms involved in this reduction of neonatal 25OHD levels are not yet known; it may be the result of different vitamin D binding proteins concentrations or a different 25OHD half-life in the neonatal age [23, 32].

Vitamin D status is related to season of birth and, as expected, 25OHD values were significantly reduced in winter and spring than in summer and autumn, regardless of supplementation and food intake.

The percentage of immigrant parents in our study was consistent with the current ethnic demographic composition of infants and children in Novara (29%). Therefore, the present study is representative of the Piedmont population.

Our results show that few migrant mothers are supplemented with vitamin D during pregnancy, especially in the last month, suggesting that more extensive prevention and education programs should be adopted.

Furthermore, when we analyzed the role of diet and supplementation in pregnancy and their effects on mothers and newborns, vitamin D intake through diet was (136 IU on average) under 400 IU (recommended minimum), with an intake slightly higher in migrants than Italian mothers. In Italy foods are not supplemented with vitamin D and the consumption of foods with high content of vitamin D is unusual.

All mothers supplemented with vitamin D had higher values of 25OHD and the same was true for their babies. These results show a strong role for supplementation in increasing 25OHD levels and also that supplementation at 400 IU/die is insufficient to provide an appropriate vitamin D status in pregnant women and their offsprings. In fact, over the 50% of subjects who had declared taking vitamin D, failed to achieve optimal serum 25OHD concentrations. Vitamin D deficiency in pregnancy in spite of taking supplementation may be as a result of an insufficient dose of vitamin D contained in multivitamins in prenatal supplements many of which contain only 400 IU or 500 IU, as previously shown in other studies [33, 34]. In particular, very few migrant mothers and newborns reached sufficient 25OHD levels despite the supplementation probably due to the contribution of both environmental factors and genetic background. This finding suggests that the migrant population is particularly at risk of deficiency and may need higher doses of supplementation. Previous studies have shown that vitamin D supplementation of 600 UI/die leads to vitamin D insufficiency in >50% of cases [33].

Recently, an increasing number of studies have shown that higher doses of vitamin D supplementation are safe and effective in pregnant women. [35–38]. However, the debate about the correct dose is ongoing and IOM recommends a dose of 400–600 IU/day in all pregnancies [14]. Moreover, some studies have shown a large inter-individual difference in response to treatment with identical doses of vitamin D suggesting that common genetic variants implicated in vitamin D homeostasis may affect the response to sun exposure or to vitamin D supplementation [39].

The relationship between 25OHD levels, ethnicity and supplementation in pregnancy suggests a role for genetic determinants for vitamin D status and stresses the importance of vitamin D integration in preventative strategies.

Our findings also underline the importance of certain risk factors in causing vitamin D deficiency such as immigration, dark skin, and lack of vitamin D supplementation during the third trimester of pregnancy. Furthermore, as maternal vitamin D deficiency status affects the newborn, it may be considered as a risk factor for other vitamin D-related disorders in these newborns. It is therefore important to correct the deficiency in utero with higher doses in those at risk of serious deficiency and to closely follow these infants re the early detection of diseases related to lack of vitamin D.

Knowledge of a generalized vitamin D deficiency and of related risk factors may lead to improving education and to more effective public health initiatives.

We did not investigate lifestyle variable such as clothing, outdoor sports activities and socio-economic status of the population and used two different assays re vitamin D levels in newborns and mothers.

In conclusion, vitamin D insufficiency in pregnancy is widely present in Piedmont and it is related to vitamin D status in newborns. We identified the clinical and anamnestic characteristics of more severe 25OHD deficit, e. g., immigration, phototype and lack of supplementation in pregnancy. A prevention program in Piedmont should urgently be considered. Vitamin D supplementation should be part of any preventative health care policy in populations at risk for rickets and others diseases related to its deficiency. Further investigations of the incidence in migrants and in their offsprings will offer a unique opportunity to understand the etiological role of 25OHD deficiency as an environmental factor.

## Supporting Information

S1 STROBE Checklist (DOC)
