# Supplementary material for: High Prevalence of Vitamin D Deficiency in Native versus Migrant Mothers and Newborns in the North of Italy: A Call to Act with a Stronger Prevention Program
Source: PLoS One. 2015 Jun 11;10(6):e0129586. doi: 10.1371/journal.pone.0129586 (PMC4466139; doi:10.1371/journal.pone.0129586)
Supplement: S1 STROBE Checklist — (DOC) [file pone.0129586.s001.doc]

STROBE checklist

**Title and abstract**

*1a. Indicate the study’s design with a commonly used term in the title or the abstract*

This has been done in both subsections. The study was a cross sectional population based study.

*1b. Provide in the abstract an informative and balanced summary of what was done and what was found*

This has been done with explanation of main outcomes and principal results (pag 2).

**Introduction**

*2. Background/rationale. Explain the scientific background and rationale for the investigation being reported*

This has been done. See pag. 3.

*3. Objectives. State specific objectives, including any prespecified hypotheses*

This has been done. See last paragraph pag.3

.

**Methods**

*4. Study design. Present key elements of study design early in the paper*

This has been done (pag. 4-5)

*5. Setting. Describe the setting, locations, and relevant dates, including periods of recruitment, exposure, follow-up, and data collection*

All these data have been included in the section “subjects and methods” (pag. 4-5)

*6. Participants. Cross sectional study*—*Give the eligibility criteria, and the sources and methods of selection of participants*

All these data have been included in the section “subjects and methods”. A flowchart of the enrolled subjects was performed ( Figure 1)

*7. Variables. Clearly define all outcomes, exposures, predictors, potential confounders, and effect modifiers. Give diagnostic criteria, if applicable*

All these data have been included (pag. 4-5)

*8. Data surce/measurements. For each variable of interest, give sources of data and details of methods of assessment (measurement). Describe comparability of assessment methods if there is more than one group*

All these data have been included (pag. 4-5)

*9. Describe any efforts to address potential sources of bias*

Possible bias have been described in the “subjects and methods “ (pag. 5) and have been stressed in study limitations (pag 10).

*10. Study size. Explain how the study size was arrived at*

These data have been included in the subsection “subjects and methods”(pag 4-6).

*11. Explain how quantitative variables were handled in the analyses. If applicable, describe which groupings were chosen and why*

How quantitative variable were handled was explained in the subsection “subjects and methods “ (pag.5). The choice of subgroups was also explained in the section “subjects and methods” (pag 5).

*12. Statistical methods (a-e).*

All information have been provided in the section “subjects and methods” (pag.6).

**Results**

*13. (a) Report numbers of individuals at each stage of study—eg numbers potentially eligible, examined for eligibility, confirmed eligible, included in the study, completing follow-up, and analysed. (b) Give reasons for non-participation at each stage(c) Consider use of a flow diagram*

All these data have been included in results (pag.6) and in Figure 1 (Flowchart showing subjects in study).

*14. Descriptive data. (a) Give characteristics of study participants (eg demographic, clinical, social) and information on exposures and potential confounders (b) Indicate number of participants with missing data for each variable of interest (c)*

All these data have been included in results. Follow-up time was not described because of the cross sectional nature of the study.

15. *Outcome data. Cross sectional study—Report numbers of outcome events or summary measures*

*Outcome events* have been summarized in the text.

16. *Main results. a*) Give unadjusted estimates and, if applicable, confounder-adjusted estimates and their precision (eg, 95% confidence interval). Make clear which confounders were adjusted for and why they were included. (*b*) Report category boundaries when continuous variables were categorized. (*c*) If relevant, consider translating estimates of relative risk into absolute risk for a meaningful time period

All these data have been included in results and tables.

17. *Other analyses. Report other analyses done—eg analyses of subgroups and interactions, and sensitivity analyses*

All results have been represented in univariate and multivariate analysis.

**Discussion**

18. *Key results. Summarise key results with reference to study objectives*

Key results have been summarised (see pag. 8). Each objective of the study have been discussed in a separate point respecting the order of introduction and results.

19. *Limitations. Discuss limitations of the study, taking into account sources of potential bias or imprecision. Discuss both direction and magnitude of any potential bias*

Limitations have been discussed at pag. 10 before the conclusion subsection.

20. *Interpretation. Give a cautious overall interpretation of results considering objectives, limitations, multiplicity of analyses, results from similar studies, and other relevant evidence*

A cautious interpretation considering other studies has been given for each specific aim.

21. *Generalisability. Discuss the generalisability (external validity) of the study results*

Generalisability has been discussed at each specific point.

**Other information**

22. *Funding. Give the source of funding and the role of the funders for the present study and, if applicable, for the original study on which the present article is based*

Funding has been described in a specific point at pag. 11.
